# Supplementary material for: Age-related changes in diffuse optical tomography sensitivity profiles in infancy
Source: PLoS One. 2021 Jun 8;16(6):e0252036. doi: 10.1371/journal.pone.0252036 (PMC8186805; doi:10.1371/journal.pone.0252036)
Supplement: S1 File — (DOCX) [file pone.0252036.s001.docx]

**Supporting Information for “Age-related Changes in Diffuse Optical Tomography Sensitivity Profiles in Infancy”**

**Method**

**MRI Segmentation**

Each head MRI volume was segmented into 9 or 10 media types: gray matter (GM), white matter (WM), cerebrospinal fluid (CSF), non-myelinated axons (NMA), other brain matter, skin, skull, air, eyes, and other inside skull material. The FSL FAST procedure [1] was used to segment the T1-weighted images into GM, WM, or other matter (OM). The GM regions was further separated into gray matter and non-myelinated axons for infants 12 months of age or younger by using the pattern of GM/WM in the average MRI template from two-year-olds as a probability map and identifying participant GM as probable GM or NMA. The CSF was identified in the T2W images using a threshold procedure. The CSF was removed from the materials from the FAST procedure, with the remainder defined as GM, WM, NMA or other inside skull material. The BETSURF procedure [2, 3] was used with the extracted brain, T1W and T2W volumes, to identify skull and scalp regions. The nasal cavity and eyes were identified manually using MRIcron [4, 5]. Finally, any other matter inside the head volume not defined as above was defined as “other inside skull material”. This generally was in the region of the neck and consisted primarily of muscle and secondarily of spinal bone.

**Mesh Generation**

We generated and dense “segmented FE mesh” (also see Method in the Main Text). Figure 1 shows the mean numbers of nodes and elements for the dense meshes across age groups. The average number of nodes was 301263, 417523, 440384, for the infants, children, and adults, respectively; average number of elements was 1,752,221, 2,449,375, and 2,582,646; and average tetra volumes were 21, 22, and 27 cubic mm. The change in node and element size reflect increases in head size over these ages.

**10-10 Electrodes Placement**

The 81 10-10 electrode locations were constructed based on the “unambiguously illustrated 10-10 system [6]. We divided the electrode positions to six groups for visualization purposes. The Cz was located at the intersection of the front-to-back central curve (Nz to Iz) and the left-to-right central curve (LPA to RPA). The “z” electrodes were placed at the 10% intervals on the Nz-Cz-Iz central curve (group 1). The LPA-RPA central curve was divided in 10% increments to set electrodes T7 to T8. From Nz to LPA to Iz, the N1, I1, and the “9” electrodes (e.g. AF9 and PO9) were identified in 10% intervals (group 2), and likewise for the N2, I2, and the “10” electrodes (e.g. AF10 and PO10) set on the right hemisphere (group 3). The curve Fpz to T7 to Oz was divided in 10% increments to generate Fp1, O1, and the “7” electrodes (group 4), and the same method was applied to identify Fp2, O2, and the “8” electrodes on the right hemisphere (group 5). Lastly, the “z”, “7” and “8” locations were used to define “1” and “2”, ‘3” and “4”, and “5 and “6” electrode locations (group 6).

**Virtual Channel Construction**

We constructed source-detector (S-D) channels from 10-5 electrode locations. The pool of channels was used to evaluate channel DOT sensitivity as a function of S-D separation distances. The target separation distances were 10mm, 15mm, 20mm, 25mm, 30mm, 35mm, 40mm, 45mm, 50mm, 55mm, and 60mm. We selected channels for each target separation distance by using a distance range that was formed by padding a small number around the target separation distance (e.g. a range of 29.2mm to 30.8mm for the target separation distance of 30cm). The ranges for target separation distances by age groups were presented in Table 1. The mean numbers of channels selected for each target separation distance by age groups were shown in Figure 2.

**Results**

**S-D Channel DOT Sensitivity Profiles by Source-Detector Separation Distances**

There were age-related differences in between-channel variance of the sensitivity profiles across separation distances. Figure 3A and 3B show examples of the sensitivity profile by channels (individual lines) from all age groups at 20mm and 50mm separation distance, respectively. Figure 4 presented the S-D Channel DOT fluence (averaged across channels with the target separation distance) as a function of sampling depth at 20mm (Figure 4A), 30mm (Figure 4B), and 50mm (Figure 4C) separation distances. The line graph presentations are displayed in the Main Text Figure 5B, 5C, and 5D. The bar charts presented in Figure 4 highlighted age-group differences at each interval of sampling depth. At 20mm separation distance, age-group differences were more visible at 10mm to 50mm sampling depth. The age-related differences in S-D Channel DOT fluence values by sampling depth increased as the separation distance increased to 30mm and 50mm especially at shallower sampling depth.

We quantified the variance by computing standard errors of the S-D Channel DOT fluence from channels with the target source-detector separation distance. Figure 6B in the Main Text, SI Figure 5 and Figure 6 present the standard errors as a function of sampling depth by individual age groups at 20mm, 30mm, and 50mm separation distances, respectively. The variance increased with separation distances for infants but remained relatively stable for children and adults. The increase in variance was greater for infants between 6 months to 2 years. The findings are also discussed in the Main Text.

We examined the shape of the S-D Channel DOT sensitivity profile by quantifying the half-width half-maximum (HWHM) location for each S-D Channel DOT fluence distribution. Main Text Figure 7A and SI Figure 7 present the HWHM location as a function of the source-detector separation distance (Main Text) or age groups (SI). Both figures show that the HWHM increased as the separation distance increased for all age groups. The mean HWHM locations for all separation distances were greater in the youngest infants (2-week through 4.5 month old age groups), children and adults, and at smaller levels in the 6-month-to-12 month ages (Figure 7A and 7B solid line blue color) and the 15-month to 2-year-old ages.

A.


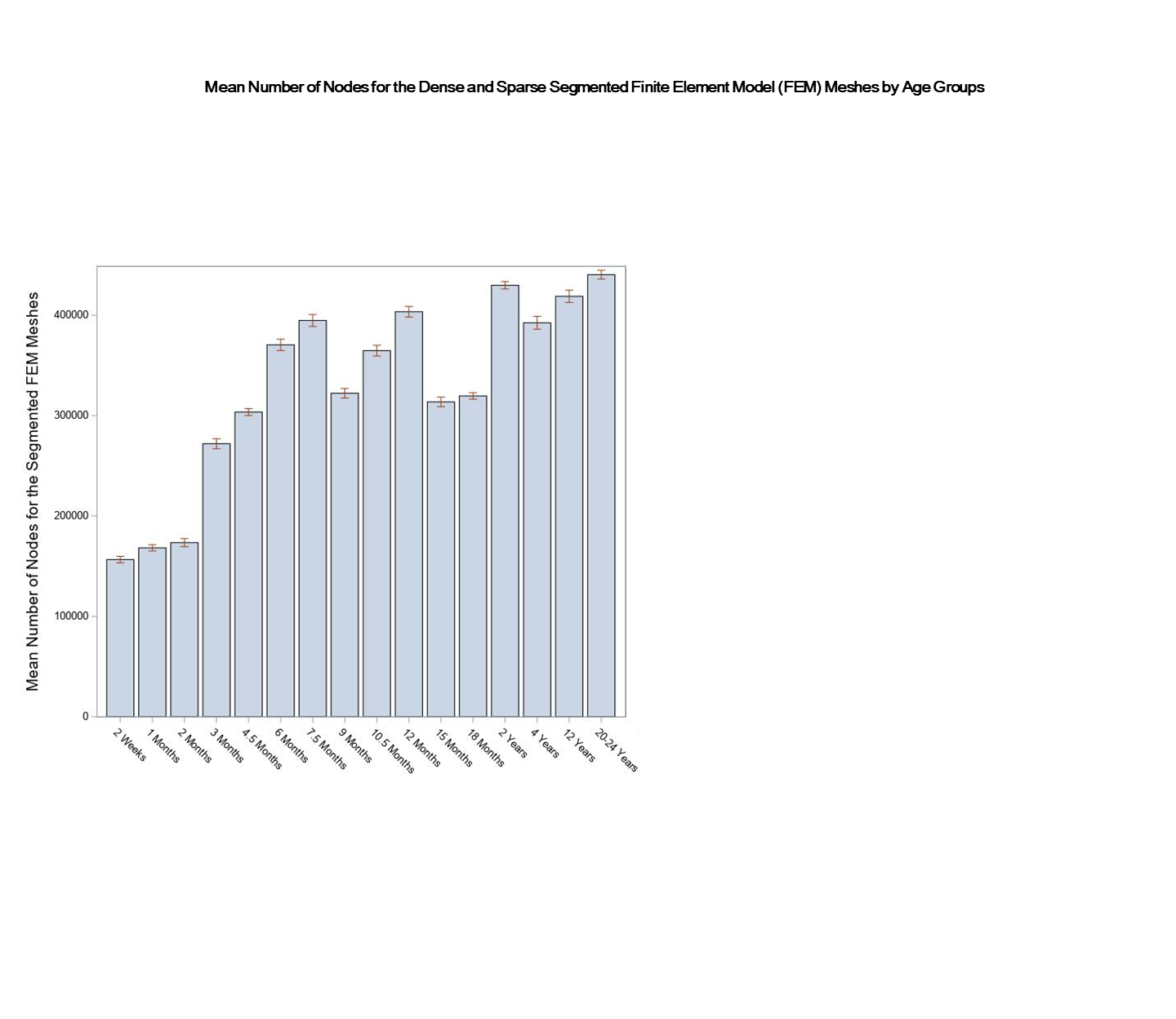


B.


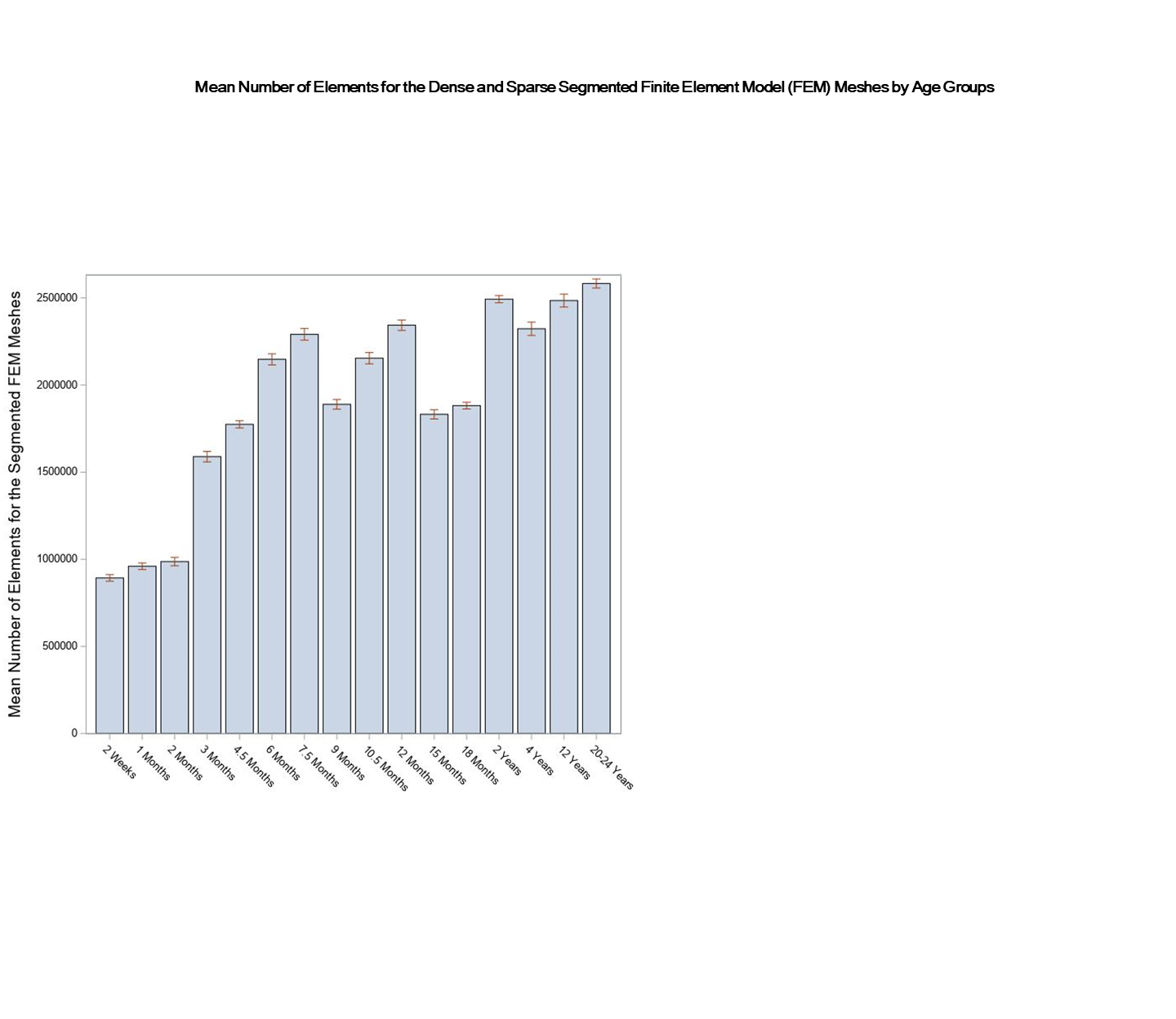


**Fig** 1. Mean number of nodes and elements for the dense finite element (FE) mesh by age groups.


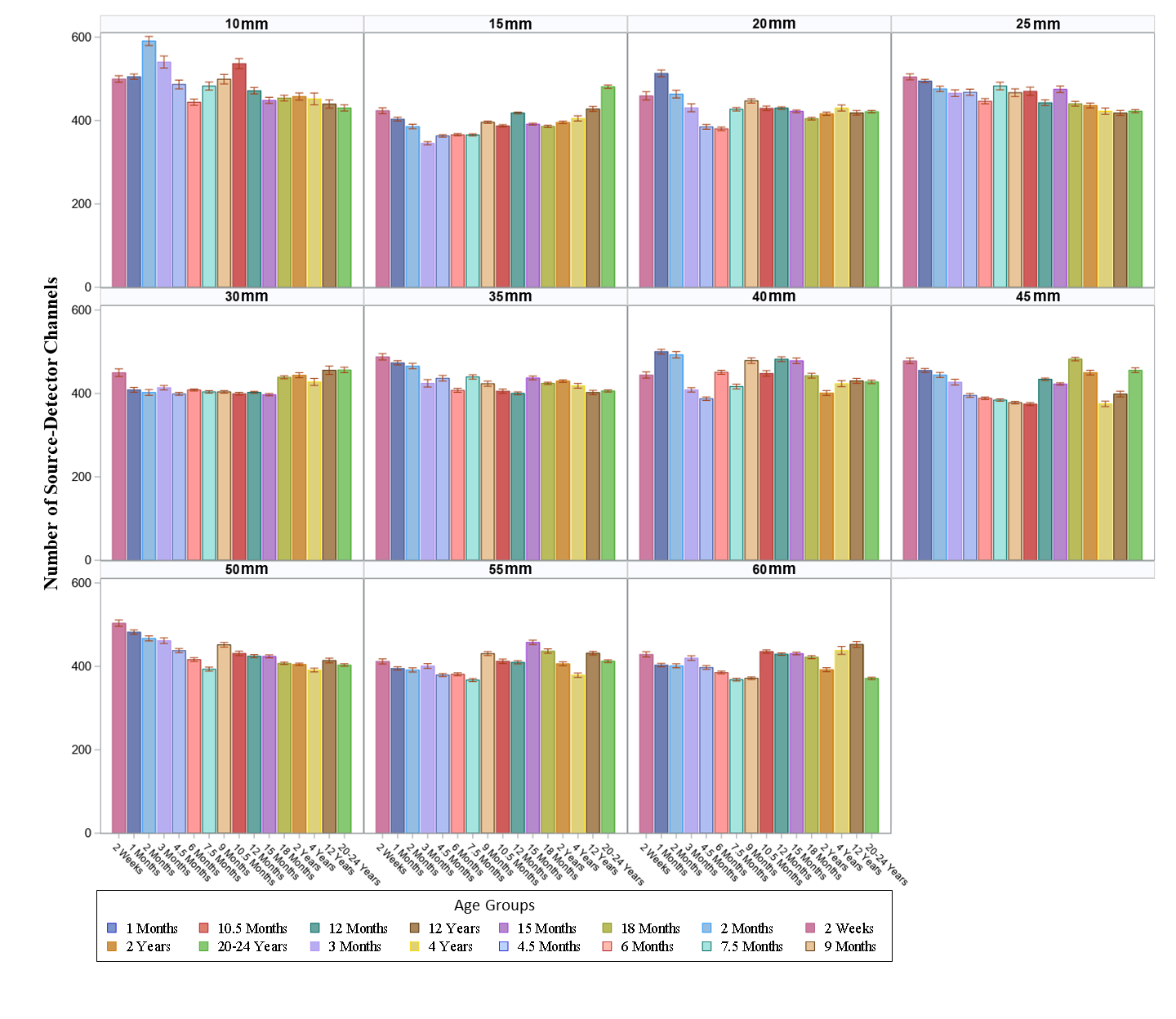


**Fig** 2. Mean numbers of source-detector channels selected for the target separation distances by age groups. The channels were formed by 10-5 electrode pairs. Error bars show ±1 standard error.

A.

**
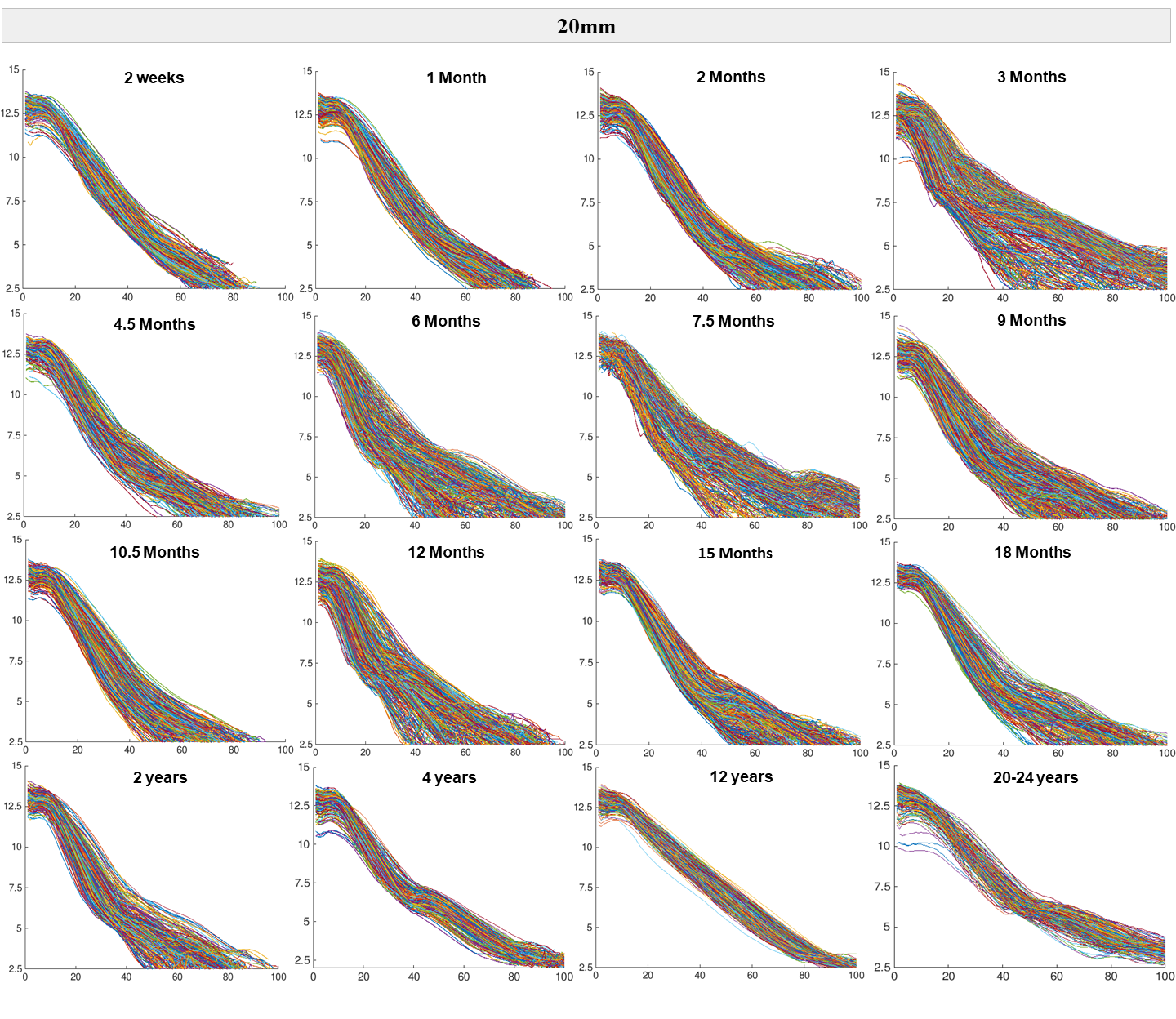
**

B.

**
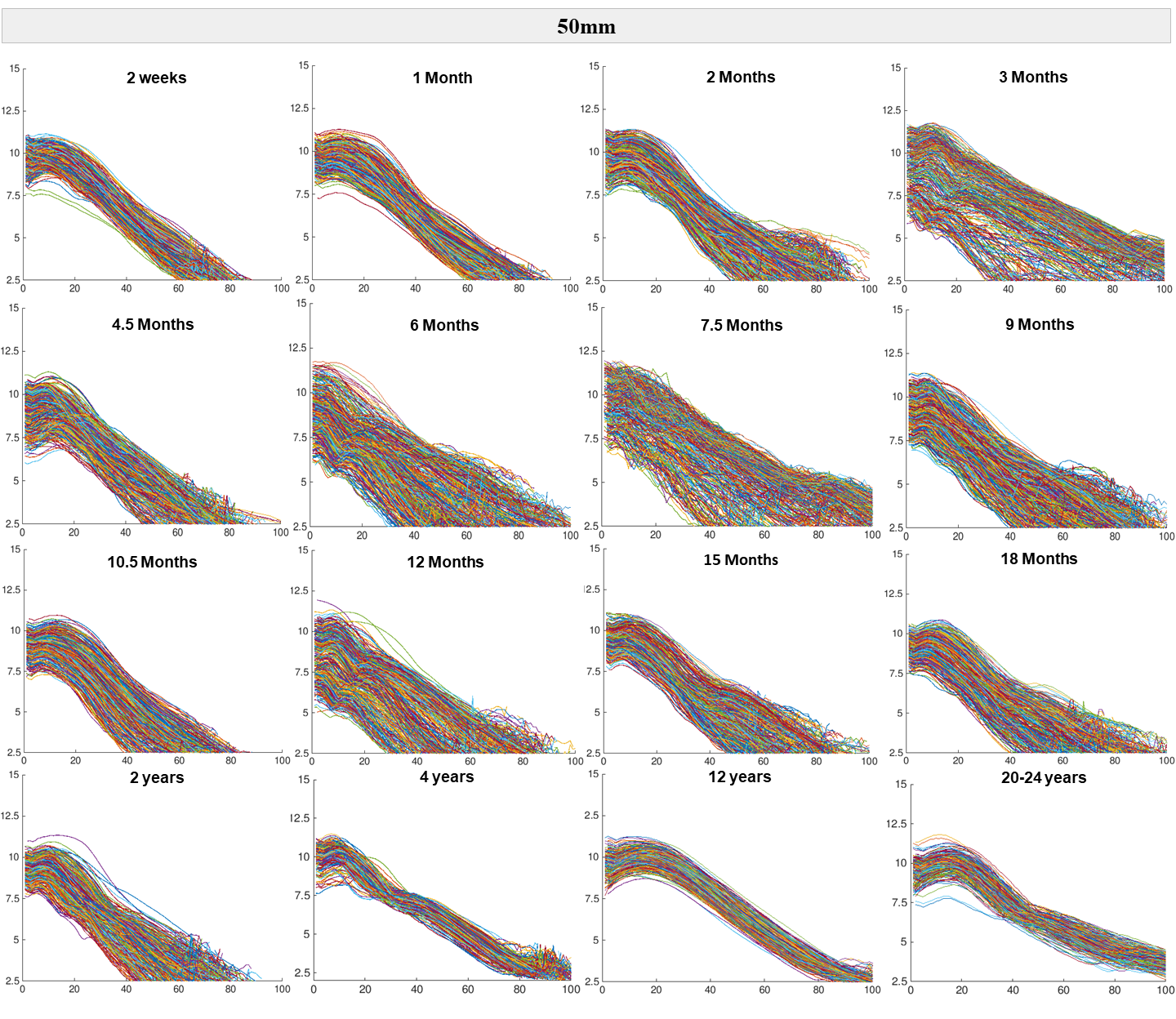
**

**Fig** **3**. Source-Detector (S-D) Channel DOT fluence sensitivity profile by channels. A. Examples of S-D Channel DOT fluence sensitivity profile by channel (colored lines) from an individual subject in each age group at 20 mm separation distance. B. Examples of S-D Channel DOT fluence sensitivity profile by channel from individual subjects at 50mm separation distance.

A.


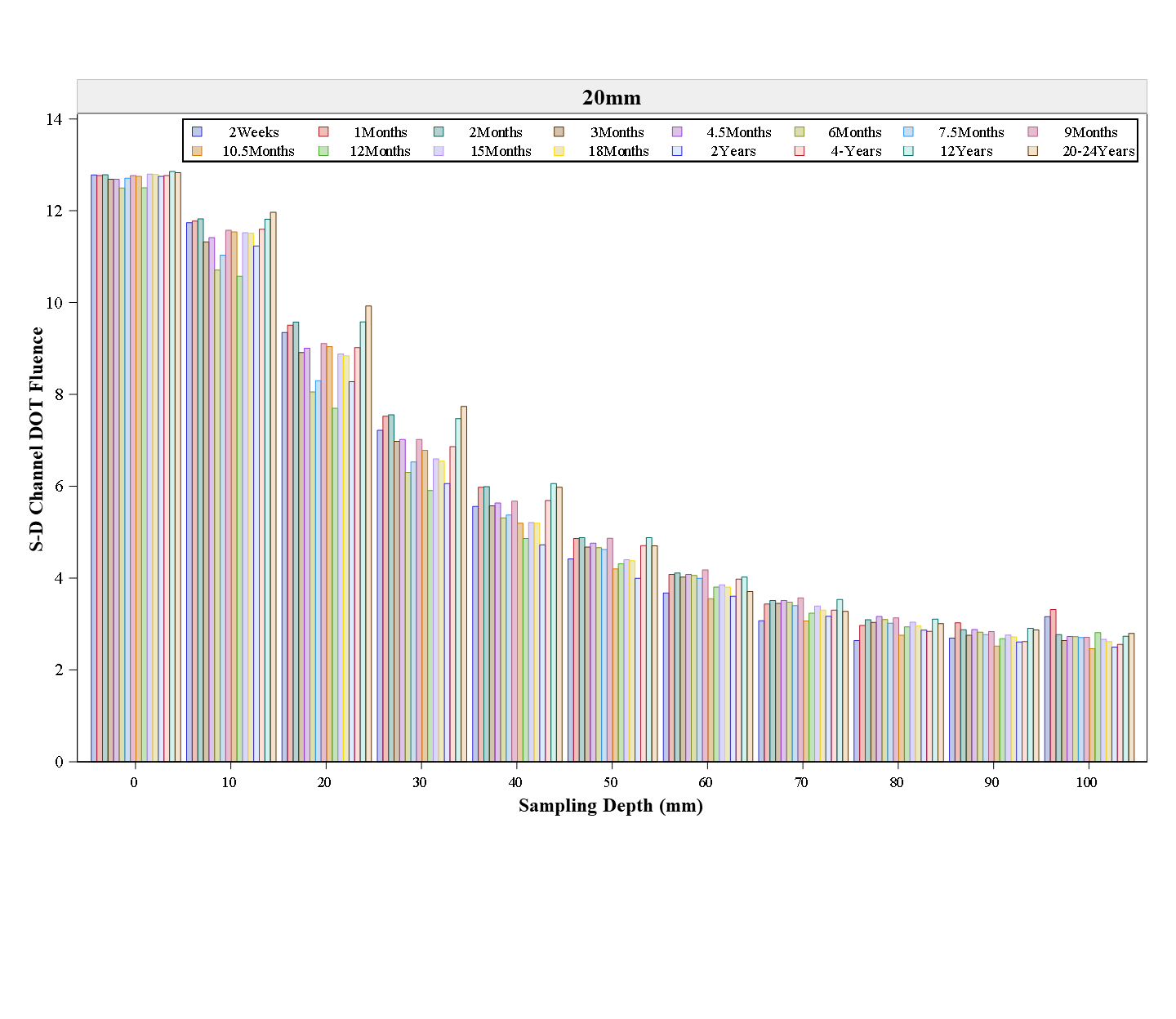


B.


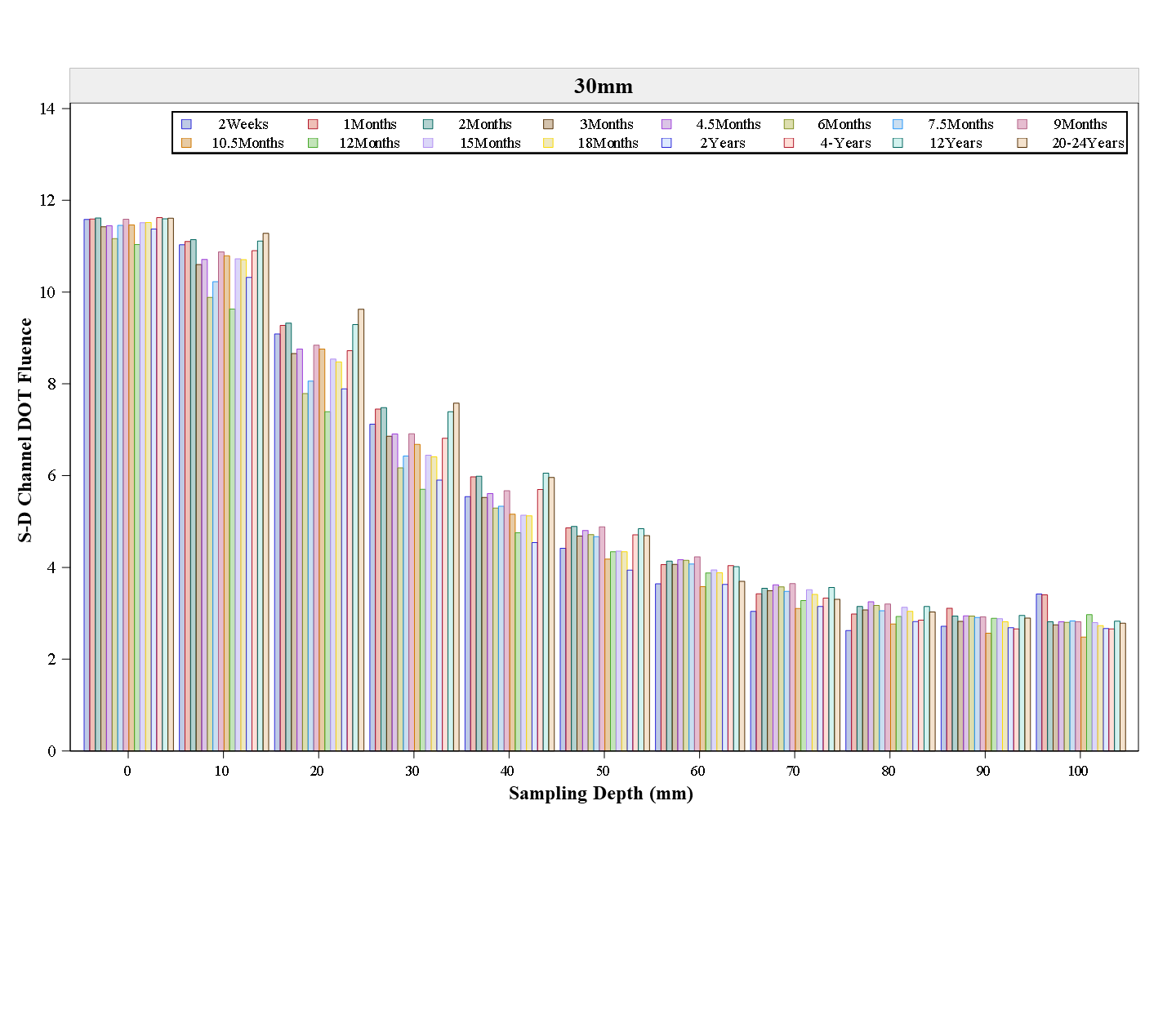


C.


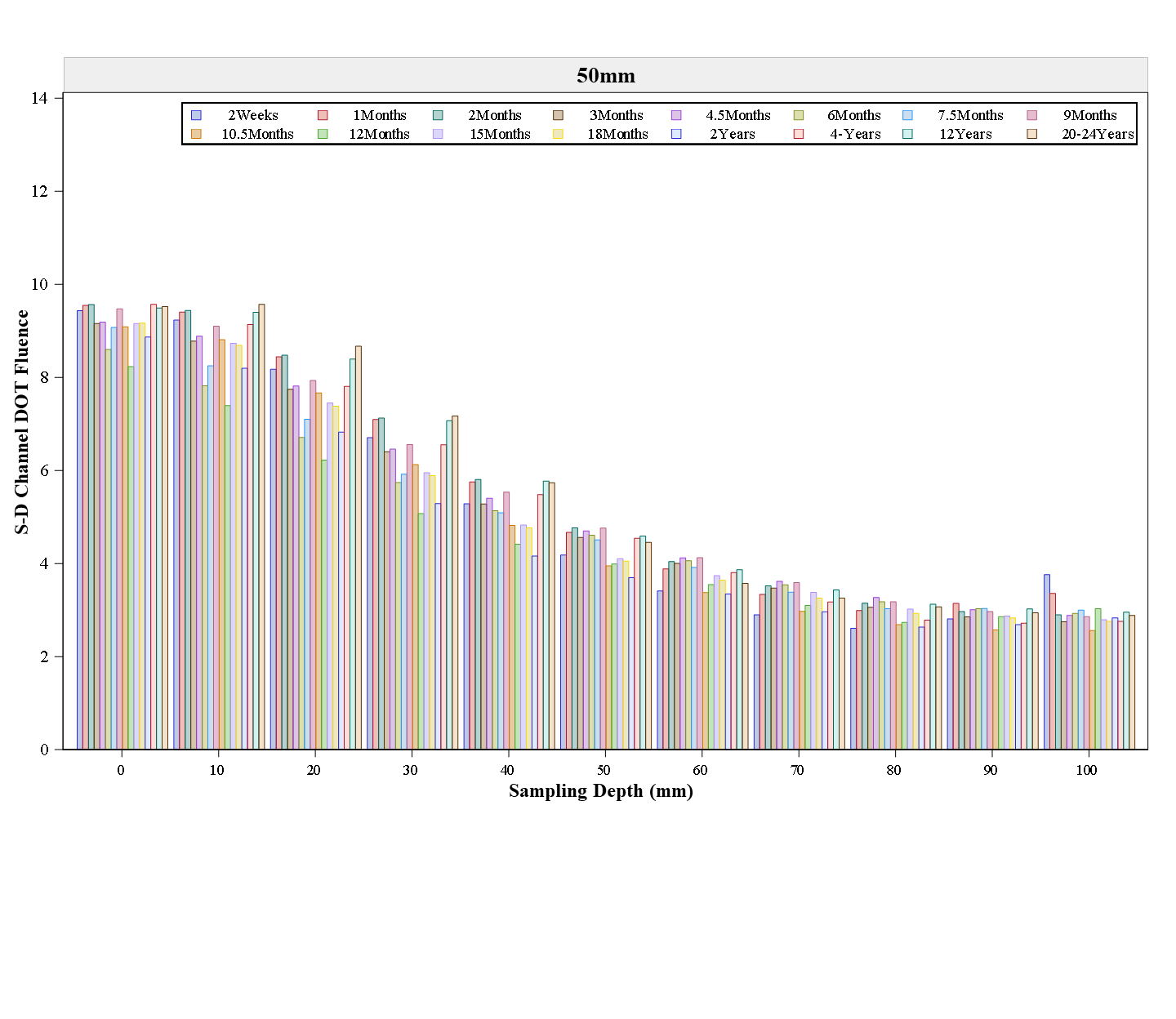


**Fig 4**. Source-Detector (S-D) Channel DOT fluence sensitivity profile by age groups at example source-detector separation distances. A. S-D Channel DOT fluence sensitivity function by individual age groups at 20 mm source-detector separation distance. B. S-D Channel DOT fluence sensitivity function by individual age groups at 30 mm separation distance. C. S-D Channel DOT fluence sensitivity function by individual age groups at 50 mm separation distance.


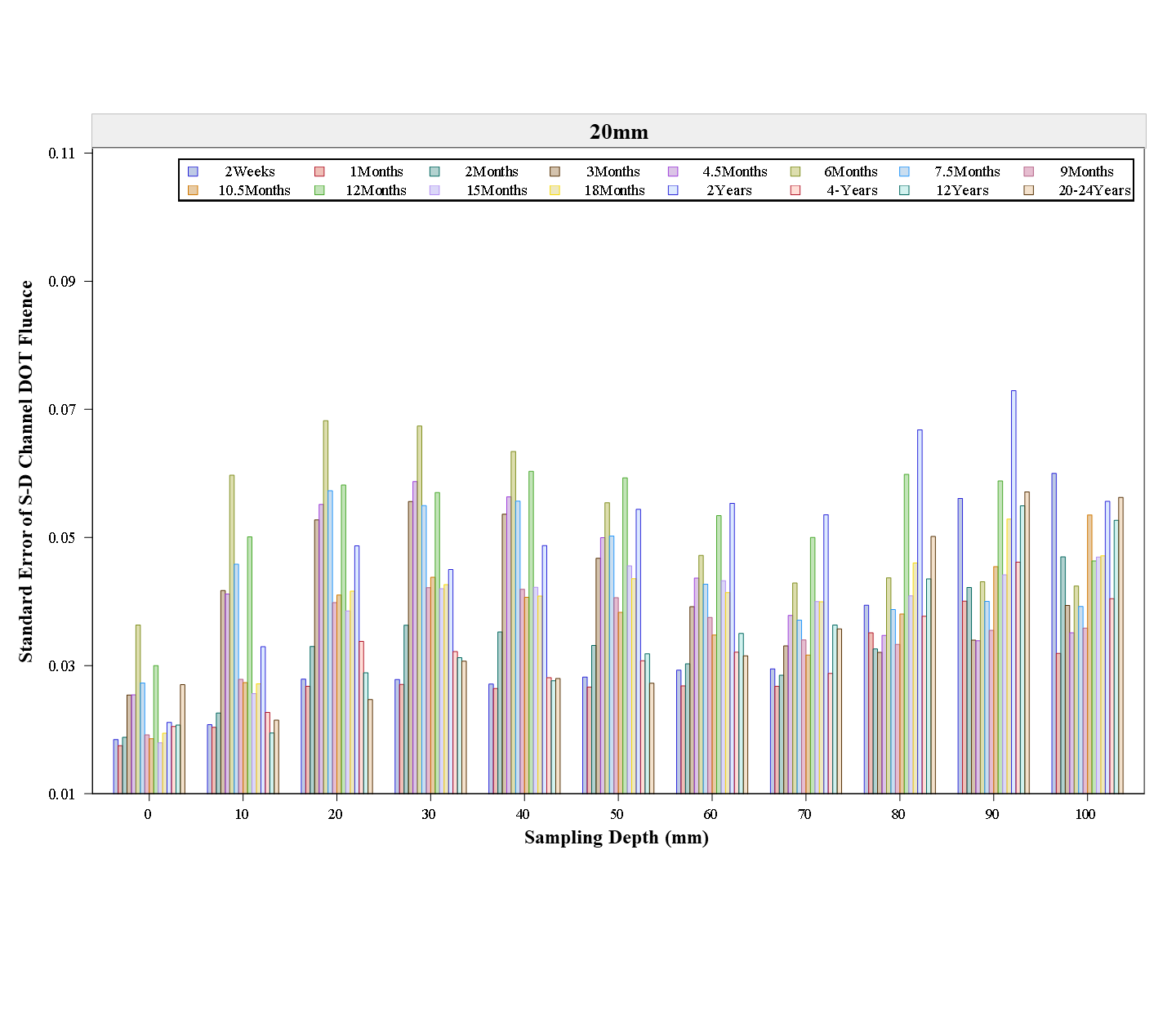


**Fig 5**. Standard error of the source-detector (S-D) Channel DOT fluence sensitivity function by individual age groups at 20 mm source-detector separation distance.

A


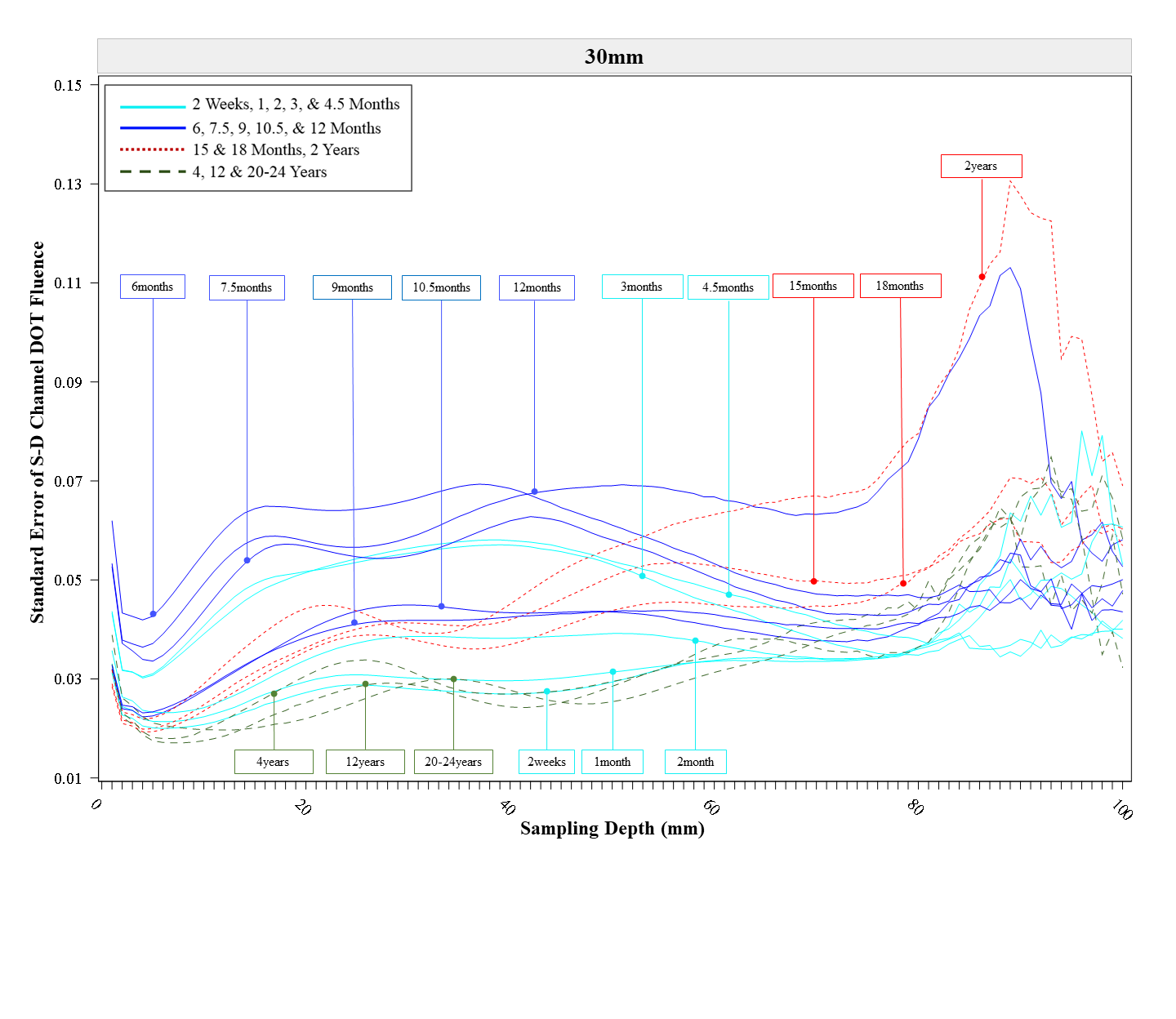


B


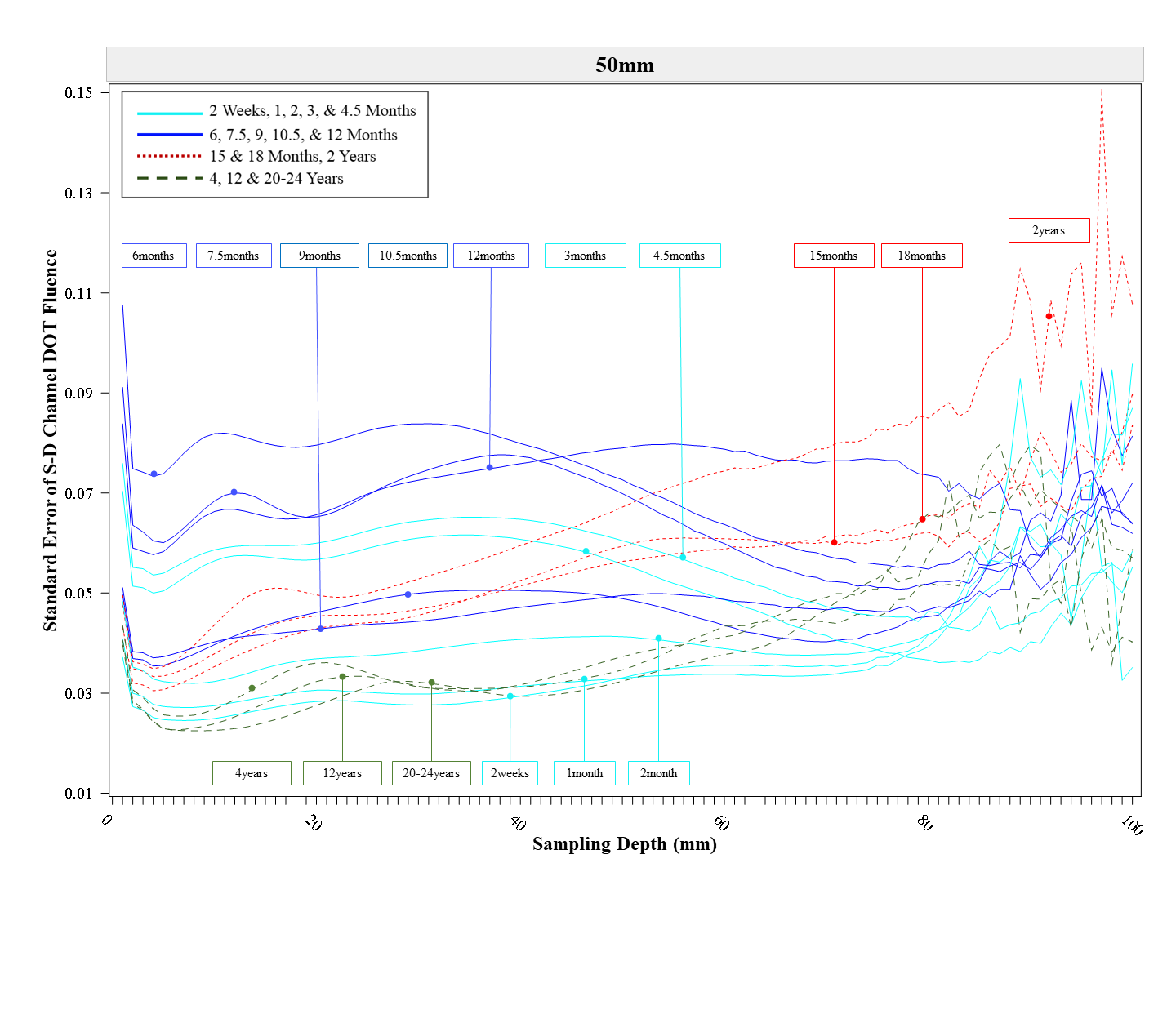


**Fig** **6**. Variance of Source-Detector (S-D) Channel DOT fluence sensitivity profile by age groups. A. Standard error of the mean S-D Channel DOT fluence value as a function of sampling depth by age groups at 30mm separation distance. B. Standard error of the mean S-D Channel DOT fluence value as a function of sampling depth by age groups at 50mm separation distance.


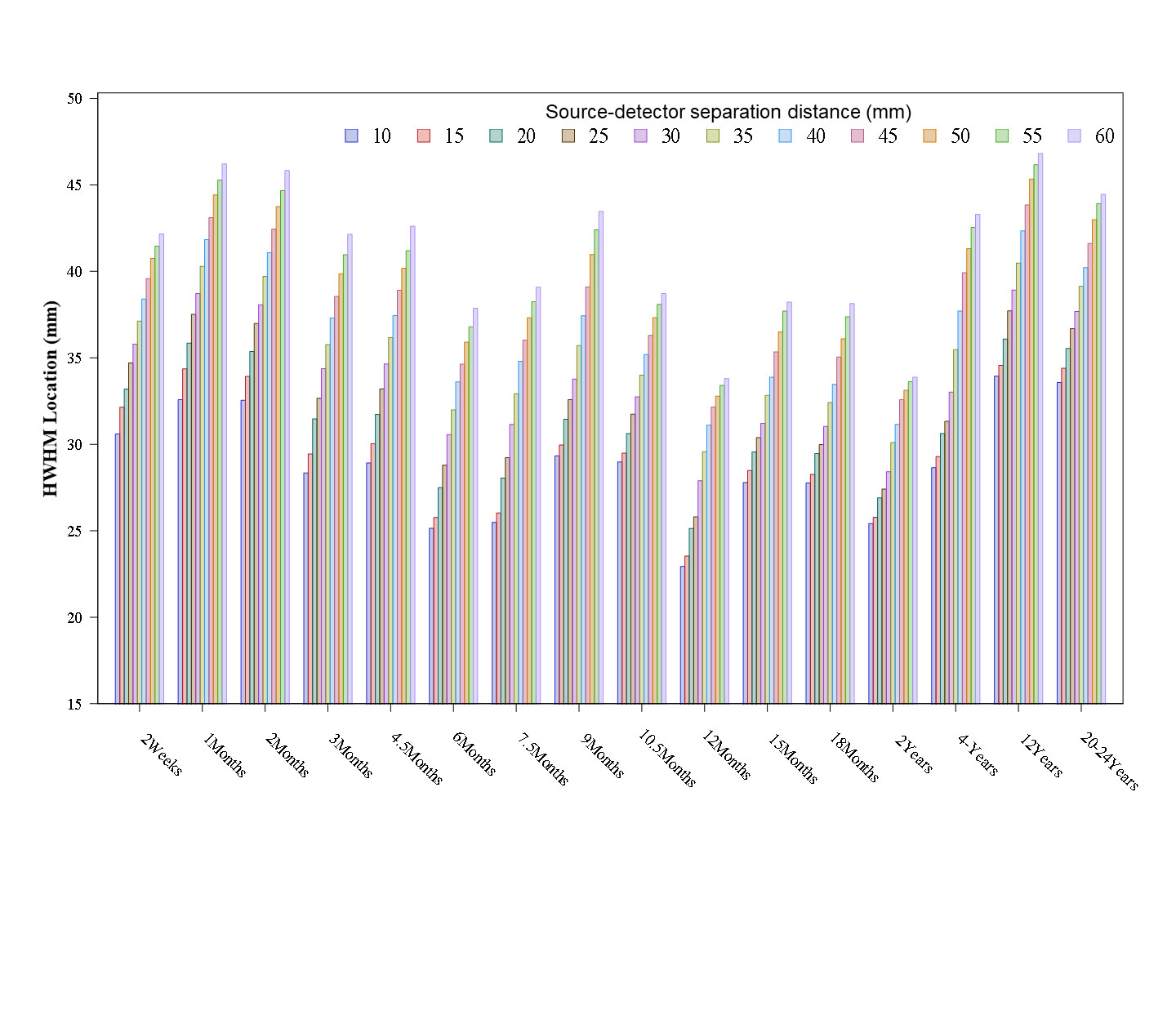


**Fig 7**. Half-width half-maximum (HWHM) locations of Source-Detector (S-D) Channel fluence as a function of age groups separately by source-detector separation distance.

**References**

1. Zhang Y, Brady M, Smith S. Segmentation of brain MR images through a hidden Markov random field model and the expectation-maximization algorithm. IEEE transactions on medical imaging. 2001;20(1):45-57.

2. Jenkinson M, Pechaud M, Smith SM. BET2: MR-based estimation of brain, skull and scalp surfaces. In Eleventh Annual Meeting of the Organization for Human Brain Mapping. 2005.

3. Smith SM, Jenkinson M, Woolrich MW, Beckmann CF, Behrens TEJ, Johansen-Berg H, et al. Advances in functional and structural MR image analysis and implementation as FSL. NeuroImage. 2004;23:S208-S19.

4. Rorden C. MRIcroGL. Retrieved from McCausland Center: <http://wwwmccauslandcenterscedu/mricrogl/>. 2012.

5. Rorden C, Brett M. Stereotaxic Display of Brain Lesions. Behavioural Neurology. 2000;12:421719.

6. Jurcak V, Tsuzuki D, Dan I. 10/20, 10/10, and 10/5 systems revisited: their validity as relative head-surface-based positioning systems. NeuroImage. 2007;34(4):1600-11.

7. Strangman G, Zhang Q, Li Z. Scalp and skull influence on near infrared photon propagation in the Colin27 brain template. NeuroImage. 2014;85:136-49.

8. Huppert TJ, Diamond SG, Franceschini MA, Boas DA. HomER: a review of time-series analysis methods for near-infrared spectroscopy of the brain. Applied Optics. 2009;48:D280-D98.

9. Custo A, Boas DA, Tsuzuki D, Dan I, Mesquita R, Fischl B, et al. Anatomical atlas-guided diffuse optical tomography of brain activation. NeuroImage. 2010;49(1):561-7.

10. Dehaes M, Kazemi K, Pélégrini-Issac M, Grebe R, Benali H, Wallois F. Quantitative effect of the neonatal fontanel on synthetic near infrared spectroscopy measurements. Human Brain Mapping. 2013;34(4):878-89.
